# A Comparative Study of Brassica Sprout Extracts on Intestinal Inflammation and Epithelial Barrier Integrity

**DOI:** 10.3390/foods15193494

**Published:** 2026-09-29

**Authors:** Stefano Piazza, Carola Pozzoli, Marco Fumagalli, Nicole Maranta, Safwa Moheb El Haddad, Lorenzo Zanardo, Luigi Alberto Marrari, Chiara Di Lorenzo, Giulia Martinelli, Enrico Sangiovanni, Mario Dell’Agli

**Affiliations:** 1 Department of Pharmacological and Biomolecular Sciences “Rodolfo Paoletti” (DiSFeB), Università degli Studi di Milano, 20133 Milan, Italy; stefano.piazza@unimi.it (S.P.); carola.pozzoli@unimi.it (C.P.); marco.fumagalli3@unimi.it (M.F.); nicole.maranta@unimi.it (N.M.); safwa.elhaddad@unimi.it (S.M.E.H.); zanardolorenzo@yahoo.it (L.Z.); chiara.dilorenzo@unimi.it (C.D.L.); enrico.sangiovanni@unimi.it (E.S.); mario.dellagli@unimi.it (M.D.); 2 Naturalsalus S.r.l., Trezzano Rosa, 20060 Milan, Italy; marrari@imospa.it

**Keywords:** *Brassica* sprouts, gut inflammation, glucoraphanin, sulforaphane, polyphenols

## Abstract

The consumption of *Brassica* spp. vegetables has been associated with antioxidant, anti-inflammatory, and prebiotic effects, mainly attributed to glucosinolates and polyphenols which are abundant in sprouts. Although broccoli sprouts are a recognized source of these bioactive compounds, other Brassicaceae sprouts remain comparatively understudied, particularly in relation to gastrointestinal disorders. In this in vitro study, hydroalcoholic extracts from red cabbage (CR) and pak-choi (PC) sprouts were compared with broccoli (BR) for their phytochemical composition, anti-inflammatory and intestinal barrier-protective activities. Caco-2 cells were stimulated with IL-1β, IFN-γ, and digested gliadin, while a Caco-2/THP-1 co-culture stimulated with LPS and IFN-γ was used to mimic intestinal permeability. CR contained the highest amounts of glucoraphanin (GFN, 22.133 ± 0.212 mg/g of extract) and sulforaphane (SFN, 9.517 ± 0.661 mg/g of extract), whereas BR showed the lowest levels. Polyphenol contents were comparable among the three extracts (3.5–4.5%). At 50 µg/mL, CR and PC extracts inhibited MCP-1 release (–37% and –48%, respectively), followed by BR (–31%), and this inhibitory effect was maintained after simulated gastrointestinal digestion. None of the extracts impaired the NF-κB-driven transcription, whereas only PC significantly enhanced the activity of Nrf-2. In the co-culture model, CR preserved epithelial barrier integrity measured by TEER and Lucifer Yellow assays (25–100 µg/mL). A PCR array showed that PC and CR (50 µg/mL) modulated genes involved in inflammation and barrier function, with a partial overlap with GFN. At the nominal *p*-value level, CR reduced CLDN1 and STAT3 transcript levels; however, these changes did not remain significant after FDR correction. These findings identify CR as a promising source of bioactive compounds associated with the preservation of epithelial barrier integrity, possibly through the modulation of selected inflammatory and barrier-related pathways. The lack of a direct relationship between glucosinolate abundance and biological activity suggests that the observed effects may depend on the overall phytochemical complexity of the extracts, including both sulfur-containing compounds and polyphenols.

## 1. Introduction

Vegetables belonging to *Brassica* spp. have a recognized role as dietary components with various health-promoting properties. Their consumption as foods or supplements has been associated with a reduced risk of several chronic diseases, including cardiovascular disease, diabetes, and cancer [1,2,3]. This evidence is supported by numerous experimental studies demonstrating antioxidant, anti-inflammatory, and antiproliferative effects [4,5,6]. The main dietary compounds to which the biological properties have been attributed are polyphenols and sulfur compounds [4,6,7,8]. The latter may reach elevated levels in meristematic tissues abundant in seeds and sprouts, which are gaining growing attention as nutraceutical products [9]. The content of sulfur compounds, such as glucoraphanin (GFN), is influenced by several agricultural variables [10]; at the same time, GFN is considered a valuable precursor of bioactive metabolites (the isothiocyanates, ITCs) generated during food processing and consumption [9].

Most published research has focused on the characterization of broccoli sprouts and seeds (*Brassica oleracea* var. *italica* Plenck.), a well-known source of ITCs [11,12]. Few comparative studies with other *Brassica* spp. have been reported, both in terms of sulfur compound content and biological properties [13,14]. Moreover, the role of edible *Brassica* spp. and their functional compounds in gastrointestinal health has been less investigated compared to their role in cardiometabolic risk.

Gut barrier dysfunction is involved in the pathogenesis of several inflammatory disorders, in which gut-organ communication may play a relevant role [15,16]. Chronic inflammatory disorders, such as celiac disease, inflammatory bowel disease (IBD), dermatitis (e.g., atopic dermatitis and psoriasis), and others, have been associated with dysbiosis and immune dysregulation [17,18]. In these contexts, dietary modulation has been recognized as a complementary approach to first-line pharmacological treatments.

*Brassica* spp. and ITCs have demonstrated promising effects in models of *H. pylori*-related gastritis [19]. Recent reviews have summarized preliminary evidence regarding the promising effects of broccoli sprout consumption in rodent models of colitis [20,21]. Several in vitro studies have supported the role of *Brassica* sprouts as potential prebiotic [22] and antioxidant foods at the intestinal level [23]. Although broccoli sprouts have been extensively investigated, comparative biological studies including other edible *Brassica* sprouts are still scarce, particularly in experimental models reproducing intestinal inflammation and epithelial barrier dysfunction. Furthermore, it remains unclear whether differences in phytochemical composition translate into distinct biological activities.

Our in vitro experiments suggested that GFN acts not only as a precursor of ITCs but also as a bioactive sulfur compound itself, due to its protective role on the intestinal epithelial barrier; moreover, sulforaphane showed anti-inflammatory properties against a pro-inflammatory insult containing gliadin [24]. Therefore, the aim of this study was to compare three edible *Brassica* sprouts in terms of phytochemical composition and biological activity using complementary in vitro models of inflammatory responses and epithelial barrier dysfunction.

## 2. Materials and Methods

### 2.1. Materials

Organic solvents such as methanol and ethanol and HPLC-grade water were purchased from VWR International (Fontenay-sous-Bois, France). Sulforaphane, sodium butyrate, gliadin, Lucifer Yellow, HBSS medium, and digestive enzymes were purchased from Sigma-Aldrich (Merck Life Science, Milan, Italy). Natural substances used as analytical and bioactive reference compounds (apigenin, glucoraphanin, and resveratrol) were purchased from PhytoLab GmbH & Co. KG (Vestenbergsgreuth, Germany) and were dissolved in DMSO at the concentration of 20 mM and stored at −20 °C until use in cell treatments.

ELISA kits and human cytokines were purchased from Peprotech, while plasmid transfection kit was purchased from Invitrogen^®^ (Thermo Fisher Scientific, Monza, Italy). Britelite^TM^ Plus reagent was purchased from Perkin Elmer (Milan, Italy).

Materials for cell culture included high-glucose DMEM; RPMI medium, Trypsin-EDTA 0.25%, fetal bovine serum (FBS), and disposable materials (Primo^®^ or Falcon^®^) were purchased from Euroclone S.p.a. (Pero, Italy); non-essential amino acids, sodium pyruvate, L-glutamine, streptomycin, and penicillin, were from Gibco^TM^ (Thermo Fisher Scientific, Monza, Italy).

### 2.2. Plant Extraction

Sprouts from broccoli (*Brassica oleracea* var. *italica* Plenck), red cabbage (*Brassica oleracea* var. *capitata* L. f. *rubra*), and pak-choi (*Brassica rapa* subsp. *chinensis* (L.) Hanelt) were cultivated for 7 days in March 2024 in a vertical farm (PiantaNatura S.r.l., Società Agricola, Mezzago (MB), Italy) and kindly donated to our laboratory. Fresh sprouts (100 g) were homogenized using a SilentCrusher (Heidolph Instruments GmbH & Co., Schwabach, Germany) in 100 mL of a water:ethanol (50:50 *v*/*v*) solvent mixture to limit myrosinase activity [25]. The plant material was then filtered under vacuum through filter paper and extracted again for 16 h with 100 mL of fresh hydroalcoholic solution (overnight, room temperature) in a glass flask, under constant magnetic agitation. After the second filtration, the extracts obtained were concentrated using Rotavapor (Heidolph Instruments GmbH); the resulting powder was obtained by freeze-drying (Edwards, 5Pascal, Trezzano, Italy) and stored at –20 °C. The extraction yields are reported in Results section.

### 2.3. Cell Culture

Human intestinal epithelial cells from colorectal adenocarcinoma (Caco-2, clone HB237; ATCC, Manassas, VA, USA) were grown for 48 h in 75 cm^2^ flasks containing high-glucose DMEM, 100 mg streptomycin, 100 units penicillin, 1% non-essential amino acids, 1 mM sodium pyruvate, 4 mM L-glutamine, and 10% FBS. Cells were maintained under a humidified atmosphere with 5% CO_2_ at 37 °C. Before reaching confluence, cells were detached with Trypsin-EDTA 0.25%, counted, and transferred to experimental supports or new flasks. To reproduce the enterocyte-like phenotype, cells were cultured on Transwell^®^ support after confluence for 17–21 days, until full differentiation was achieved [24]. This differentiation period resulted in TEER values above 700 Ω.

THP-1 human monocytic leukemia cells (ATCC, Teddington, UK) were cultured in 75 cm^2^ flasks containing RPMI medium, 100 mg streptomycin, 100 units penicillin, 4 mM L-glutamine, and 10% FBS. After 48 h or 72 h, the cell suspension was centrifuged, counted, and transferred to experimental supports or new flasks.

### 2.4. Cell Treatments

For the biological investigation, Caco-2 cells were seeded in 24-well plates (3 × 10^4^/well) for 48 h, then stimulated with a combination of IL-1β (10 ng/mL), IFN-γ (10 ng/mL), and in vitro-digested gliadin (Ga) (1 mg/mL) for 6 h or 24 h, following previously established conditions [24]. The co-culture model (THP-1/Caco-2) was established by seeding confluent Caco-2 cells (3 × 10^5^/well) in the upper Transwell^®^ insert; during their differentiation period (17–21 days), THP-1 cells seeded in 12-well plates (2 × 10^5^/well) were exposed to PMA (25 nM) to allow differentiation into adherent macrophages within 48 h [26]. On the day of treatment, wells containing differentiated Caco-2 cells were placed onto 12-well plates containing THP-1 macrophages stimulated with LPS (100 ng/mL) and IFN-γ (10 ng/mL) for 24 h or 48 h [24]; the procedure regarding THP-1 stimulation was also used in experiments involving macrophages separately. For all experiments, cells were concomitantly exposed to solutions of plant extracts or reference anti-inflammatory compounds diluted in FBS-free DMEM medium, namely the flavonoid apigenin (20 μM) or sodium butyrate (2 mM) selected based on previously published studies [27]. Plant extract stock solutions were prepared in water:DMSO (50:50) at 20 mg/mL, whereas reference compounds were dissolved in DMSO (20 mM). The vehicle (DMSO) was always normalized in cell treatments with a final concentration equal to or below 0.2%.

### 2.5. Cell Viability

The morphology of cells was observed by light microscopy inspection at the beginning and at the end of each experiment. Cell viability was measured using the 3,4,5-dimethylthiazol-2-yl-2-5-diphenyltetrazolium bromide (MTT) assay [24]; absorbance values were considered acceptable when they were greater than 80% of those of untreated controls. Briefly, at the end of the experiments, treatment was stopped by removing the medium, and an MTT solution (200 μg/mL in PBS) was added to the cells. Purple formazan salts were then solubilized with an isopropanol:DMSO (90:10 *v*/*v*) solution after 15–30 min of incubation and the absorbance was read at 550 nm (VICTOR X3; PerkinElmer, Milan, Italy).

### 2.6. Evaluation of Chemokine Release

Chemokines involved in intestinal inflammation (IL-8, CXCL-10, and MCP-1) were quantified in cell culture media using ELISA, according to previously described procedures [24,27] and the manufacturer’s instructions. Briefly, EIA/RIA plates (Merck Life Science, Milan, Italy) were coated with capture antibodies specific for the target proteins and incubated overnight at room temperature. Subsequently, 100 μL of either cell culture medium or standard solution was added to each well, using a calibration curve ranging from 0 to 1500 pg/mL. Following incubation, chemokine concentrations (pg/mL) were determined by adding a biotinylated primary antibody followed by avidin-horseradish peroxidase enzyme. The resulting colorimetric reaction was developed using 2,2′-azino-bis(3-ethylbenzothiazoline-6-sulfonic acid) (ABTS) as the substrate (Merck Life Science, Milan, Italy). The absorbance was measured at 405 nm (green) using a VICTOR X3 spectrophotometer (PerkinElmer, Milan, Italy). Chemokine concentrations were expressed as mean ± SEM (pg/mL) of at least three independent experiments and reported as percentages relative to the stimulated control, which was arbitrarily set at 100%.

### 2.7. Measurement of Epithelial Integrity

The epithelial barrier integrity was evaluated in the THP-1/Caco-2 co-culture model by transepithelial electrical resistance (TEER) and Lucifer Yellow (LY) paracellular permeability, as previously described [24]. The compounds under investigation were added to the apical side to reproduce dietary exposure. At the beginning of each experiment (t0), three concordant TEER measurements (Ω) were obtained using an EVOM3 device (WPI, Sarasota, FL, USA), with cut-off value above 400 Ω. The variation in TEER values (∆Ω) was calculated at the end of the experimental period (t48 h) according to the following equation: ∆Ω = Ωt48 h − Ωt0. The results were normalized to the stimulated condition, which was arbitrarily set to 0. Data were expressed as the mean of normalized ∆Ω ± SEM.

In separate experiments, paracellular permeability was assessed by measuring LY diffusion. After a single wash with PBS, 500 μL of LY solution (10 μg/mL in HBSS) was added to the apical compartment, while 1 mL of HBSS was added to the basolateral compartment. After 1 h of incubation, 200 μL of medium was collected from the basolateral compartment and transferred to a black plate, and LY fluorescence was measured using a VICTOR X3 system (PerkinElmer, Milan, Italy). Data were expressed as mean fluorescence ± SEM from at least three independent experiments and normalized to the stimulated control, which was arbitrarily set to 100%.

### 2.8. Luciferase Assay and Transcription Factors

Caco-2 cells were transiently transfected with plasmids containing response elements for Nrf-2 (200 ng per well) or NF-κB (100 ng per well). The activity of the two transcription factors was assessed after 6 h of treatment with the substances under investigation and pro-inflammatory stimuli (IL-1β, IFN-γ, and Ga). Resveratrol and apigenin (20 μM) were used as reference natural compounds with antioxidant and anti-inflammatory activities, respectively.

The NF-κB plasmid was kindly provided by Dr. N. Marx (Department of Internal Medicine-Cardiology, University of Ulm; Ulm, Germany), whereas the Nrf-2 plasmid was provided by O’Connell et al. [28] through the Addgene non-profit repository (#90398, Addgene, LGC Standards, Teddington, UK). Transient transfection was performed using Lipofectamine^®^ 3000 Reagent, according to the manufacturer’s instructions (Thermo Fisher Scientific, Monza, Italy) [24,27]. At the end of the treatment, cells were lysed and luciferase activity was determined following the addition of luciferin using the Britelite^TM^ Plus reagent (Revvity, Waltham, MA, USA). Luminescence was acquired using a VICTOR X3 plate reader (Perkin Elmer, Milan, Italy).

### 2.9. LC-MS/MS Analysis

LC-MS/MS analysis was performed on *Brassica* sprouts extracts before and after simulated gastro-intestinal digestion, according to previously described protocols [24,27]. Briefly, HPLC was carried out using an Exion LC^TM^ AC System (AB Sciex, Foster City, CA, USA) equipped with a vacuum degasser, a double-plunger pump, a cooled autosampler, and a temperature-controlled column oven. Mass spectrometric analysis was performed using a TripleQuad^TM^ 3500 system (AB Sciex, Foster City, CA, USA). Chromatographic separation was achieved using a Synergi 4 μm Hydro-RP 80 A LC Column 150 × 4.6 mm (Phenomenex, Torrance, CA, USA). The mobile phase consisted of 0.1% formic acid in water (A) and 0.1% formic acid in methanol (50:50, B), delivered at a flow rate of 0.600 mL/min. The injection volume was 10 μL for each sample. Mass spectrometric detection was performed in negative electrospray ionization (ESI) mode for glucoraphanin and in positive ESI mode for sulforaphane. The compound-dependent MS/MS parameters (declustering potential, entrance potential, collision energy, and collision cell exit potential) were optimized in multiple-reaction-monitoring (MRM) mode by separate infusion of the analytes. Quantification was performed using the following mass transitions: 178/114 (sulforaphane), 436/97 (glucoraphanin), using commercially available pure standards. The limit of detection (LOD) was 0.0000157 ng/μL for sulforaphane and 0.0014 ng/μL for glucoraphanin, respectively. The limit of quantification (LOQ) was 0.0000476 ng/μL and 0.0041 ng/μL, respectively. The LC–MS/MS system was controlled using AB Sciex Analyst^®^ software (version 1.7).

### 2.10. Measurement of the Antioxidant Capacity Through DPPH and ORAC Assays

The antioxidant capacity of the extracts was evaluated spectrophotometrically using the 1,1-diphenyl-2-picrylhydrazyl (DPPH) and oxygen radical absorbance capacity (ORAC) assays, as previously described [24,27].

For the DPPH assay, 150 μL of 0.005% DPPH solution in methanol was mixed with 50 μL of each prepared sample. After 30 min of incubation, absorbance was measured at 517 nm using an Enspire^®^ multimode plate reader (PerkinElmer, Milan, Italy). Radical-scavenging activity was determined using a gallic acid calibration curve ranging from 1.0 to 5.0 μg/mL (equivalent to 6 to 30 μM) and expressed as gallic acid equivalents (GAE, μM).

For the ORAC assay, 40 mM AAPH (Merck Life Science, Milan, Italy) was used to generate peroxyl radicals. An aliquot of the sprout extract stock solutions was transferred to a black 96-well plate and diluted to a final volume of 20 μL. Then, 120 μL of fluorescein solution (70 nM final concentration), previously prepared in phosphate buffer (pH 7.4, 75 mM), was added to each well. AAPH solution was then added (60 μL) to obtain a final volume of 200 μL. The plate was placed in a VICTOR X3 plate reader (PerkinElmer, Milan, Italy), and fluorescence was measured at excitation and emission wavelengths of 484 and 528 nm, respectively, every 2 min for 60 min at 37 °C after shaking. Trolox (0–120 μM) was used as a reference antioxidant compound. The area under the curve (AUC) was calculated for each extract, and the results were expressed as μM Trolox equivalents.

### 2.11. RNA Extraction and PCR Array

The expression of 42 genes involved in inflammation, gut barrier function, cellular differentiation, and oxidative stress was measured using a customized PCR RT^2^ Profiler^TM^ (QIAGEN, Hilden, Germany); 3 housekeeping genes and 3 reverse transcription/PCR positive controls were also included (Table 1). A comprehensive list of the analyzed genes, including reference sequence numbers, is reported in the Supplementary Materials (Table S2). RNA was isolated from Caco-2 cells at the end of treatment (24 h) using NucleoSpin^®^ RNA Plus extraction kit (Macherey-Nagel, Düren, Germany). Cells were homogenized and lysed in 350 μL of sample buffer, and genomic DNA was removed by centrifugation through exclusion columns at 11,000 g for 30 s. RNA in the flowthrough was subsequently bound to a silica column, while other biological interferences were removed by centrifugation at 11,000 g for 15 s. RNA samples were washed three times on the column using different washing buffers and centrifugation steps. Finally, silica membranes were dried by prolonged centrifugation at 11,000 g for 2 min, and RNA was eluted in 60 μL of RNAase-free water. RNA concentration and purity were measured using a NanoDrop ND-1000 (Thermo Fisher Scientific, Monza, Italy). For PCR analysis, cDNA was synthetized from 400 ng of RNA sample using the RT^2^ First Strand kit (QIAGEN). SYBR Green Master Mix RT^2^ reagent was subsequently added, and the resulting mixture was loaded onto a 384-well plate. Amplification and quantification were performed using a C1000^TM^ Thermal Cycler coupled with CFX384^TM^ Real-Time PCR Detection System (Bio-Rad Laboratories, Segrate, Italy).

CT values were exported to an Excel template using a cut-off value of 35, according to the manufacturer’s web portal SABiosciences guidelines (QIAGEN). The resulting dataset was then uploaded to the GeneGlobe data analysis web portal. Samples were assigned to control and test groups and normalized based on a manual selection of the most reproducible housekeeping gene, GAPDH. Fold changes were calculated using the ΔΔCT method: ΔCT values were first calculated by subtracting the mean CT of the housekeeping gene from the CT of each target gene. Subsequently, the difference between the ΔCT values of the test and control groups was calculated to obtain the ΔΔCT value. Fold changes were then calculated as 2^−ΔΔCT^. The data analysis web portal also generated scatter plots and tables reporting the Fold Changes and *p*-values.

### 2.12. Total Phenolic, Anthocyanin, and Flavonol Content

The Folin–Ciocâlteu test is widely used to measure the total polyphenol content. Briefly, 20 μL of plant extract (1 mg/mL in water) was diluted with water to 800 μL, followed by addition of 50 μL of Folin reagent and 150 μL of 5% (*w*/*v*) sodium carbonate to increase the pH, reaching a final volume of 1 mL. A standard curve of gallic acid (GA, 0–30 μg/mL) was used to compare the absorbance at 765 nm (JASCO V630 International Co., Ltd., Tokyo, Japan) after 20 min of incubation at 37 °C. Total polyphenol content was expressed as mg GA equivalents (GA eq.) per g of extract ± SD.

The Aluminum Chloride (AlCl_3_) test identifies the presence of flavonoids, with specific reference to flavonols. As previously described [29], sample extracts were diluted into methanol in comparison to quercetin (standard flavonol). Then, samples were combined with 20 μL of AlCl_3_ (10% *w*/*v*), 20 μL of CH_3_COOK (1 M), 560 μL of water, and methanol to reach the final volume of 1 mL. Blank samples were obtained by excluding the salt from the reaction mixture. A reference calibration curve (0–20 μg/mL) was made by serial dilution of quercetin and absorbance at 430 nm was read by a photometer (JASCO V630 International Co., Ltd., Tokyo, Japan). Results were then expressed as mg of quercetin eq. per g of extract ± SD.

To determine the total anthocyanin content, the extracts were diluted in 70% acidic ethanol (ethanol/water/hydrochloric acid 37%). Subsequently, sample absorbance was measured at 540 nm using a spectrophotometer (Jasco V630 International Co., Ltd., Tokyo, Japan). The concentration (c) was calculated using the Beer-Lambert law. A = ε × l × c, where A is the absorbance at 540 nm, ε is the molar extinction coefficient of cyanidin-3-glucoside in acidic ethanol (14.51 L·mol^−1^·cm^−1^), and l is the optical path length. Dilution factors were incorporated into the final calculation. The results are expressed as mg of cyanidin-3-glucoside eq. per g of extract ± SD.

### 2.13. Simulated Gastrointestinal Digestion

Gastrointestinal digestion was performed following well-established protocols. Briefly, 200 mg of plant extract was sequentially digested with reconstituted saliva (6 mL), gastric juice (12 mL, pH 2), and duodenal juice (12 mL, pH 7.2). Digestion blanks (containing only enzymes and salts) were processed in parallel to account for potential matrix interference. The resulting samples were freeze-dried (Edwards, 5 Pa, Trezzano, Italy). The specific composition of each fluid is reported in [30]. The in vitro digestion was carried out once, generating a representative batch that was subsequently dissolved in the same solvent used for the corresponding undigested sample (20 mg/mL of water: DMSO solution), aliquoted, and stored at –20 °C. In all biological assays, the digestion blank was added to the corresponding control conditions at a volume equivalent to that used for the digested extracts.

Gliadin used for inflammatory stimulation was subjected to a simplified gastrointestinal digestion protocol (peptic-tryptic digestion), as described in detail in previous publications [24].

### 2.14. Statistical Analysis

All biological data were normalized and expressed as mean ± SEM. At least three independent experiments (*n* = 3) were performed, with each sample measured in duplicate. All chemical data were normalized and expressed as mean ± SD. Data were checked for outliers (ROUT method) and normality and homogeneity of variances (Shapiro–Wilk test). Statistical analyses were then performed using one-way ANOVA followed by the Bonferroni multiple-comparison test, with each treatment compared with the corresponding stimulated control. Calculations were conducted using GraphPad Prism 9.0 software (GraphPad Software, San Diego, CA, USA), and *p* < 0.05 was considered statistically significant. Calculations concerning group differences after PCR array were assessed on a per-gene basis using Student’s *t*-tests, generating one *p*-value for each of the 48 genes analyzed by the web portal at http://www.qiagen.com/geneglobe (accessed on 17 October 2025). To account for multiple comparisons across the panel, raw *p*-values were additionally adjusted (*q*-values) using the Benjamini–Hochberg false discovery rate (FDR) procedure.

## 3. Results

### Functional Compounds in Brassicaceae Samples and Their Bioactivity

Fresh Brassicaceae sprouts were first extracted with a hydroalcoholic solvent to obtain a raw extract rich in polar dietary compounds. A noteworthy extraction yield was measured for all samples (Table 2), considering the typically high water content of fresh sprouts. The highest extraction yield was obtained for CR (2.07% *w*/*w*).

Since Brassicaceae sprouts have been investigated for their content of bioactive sulfur compounds, LC-MS analysis was performed to quantify the presence of GFN and SFN in all samples. Unexpectedly, GFN and SFN were not detected in BR; in contrast, they were present at relevant levels in PC and CR (Table 3). The latter was identified as a valuable source of GFN, reaching approximately 2% *w*/*w* of sprout extract. Moreover, compound levels were maintained after the freeze-drying process (Table S1), suggesting the potential stability of sulfur compounds in the powdered extracts.

**Figure 1:**
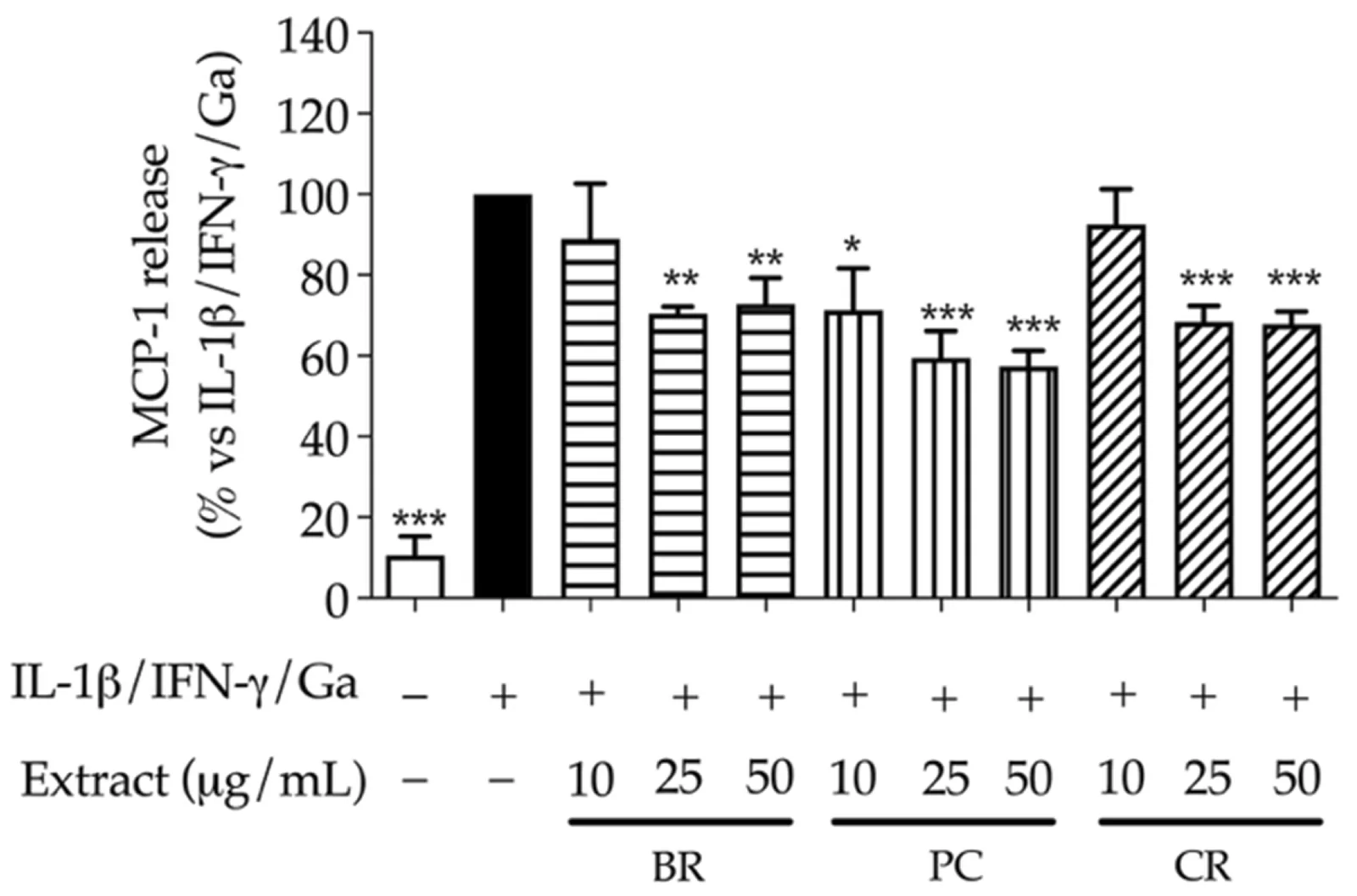
Effect of Brassicaceae sprout extracts on MCP-1 release by Caco-2 cells measured by ELISA. Cells were treated with sprout extracts and pro-inflammatory cocktail (IL-1β, IFN-γ, Ga) for 24 h. BR (broccoli), PC (pak-choi), CR (red cabbage) correspond to horizontal, vertical, and diagonal lines, respectively; unstimulated control and pro-inflammatory stimulus correspond to white and black columns, respectively. Apigenin 20 µM was used as a reference inhibitor (–65%). Data from *n* = 3 independent experiments were reported as mean of release (±SEM) vs. normalized stimulus (100%). * *p* < 0.05, ** *p* < 0.01, *** *p* < 0.001 vs. stimulus (one-way ANOVA).

Following well-established experimental settings [24], the biological activity of the sprout extracts was investigated in Caco-2 cells stimulated with a pro-inflammatory cocktail containing IL-1β, IFN-γ, and digested gliadin (Ga). Within this cocktail, gliadin was previously demonstrated to enhance the release of IL-8 (a chemoattractant for neutrophils), CXCL-10 (a chemoattractant for lymphocytes), and MCP-1 (a chemoattractant for monocytes) compared with cytokines alone. Sprout extracts were investigated at a concentration of 100 µg/mL, showing no cytotoxicity detected by the MTT assay (Figure S1). Moreover, an inhibitory activity on the release of MCP-1 was observed (Figure 1), whereas the other selected chemokines were not affected (Figure S2). At the concentration of 50 µg/mL, PC showed the best inhibitory effect (–48%), followed by CR (–37%) and BR (–30%).

Intriguingly, these results were consistent with our previous data [24], which attributed to SFN a more potent inhibitory activity on MCP-1 in comparison with the other chemokines (CXCL-8, CXCL-10). However, the amount of SFN present in sprout extracts was not correlated with their inhibitory activity, suggesting a role for other functional compounds, such as polyphenols.

**Figure 2:**
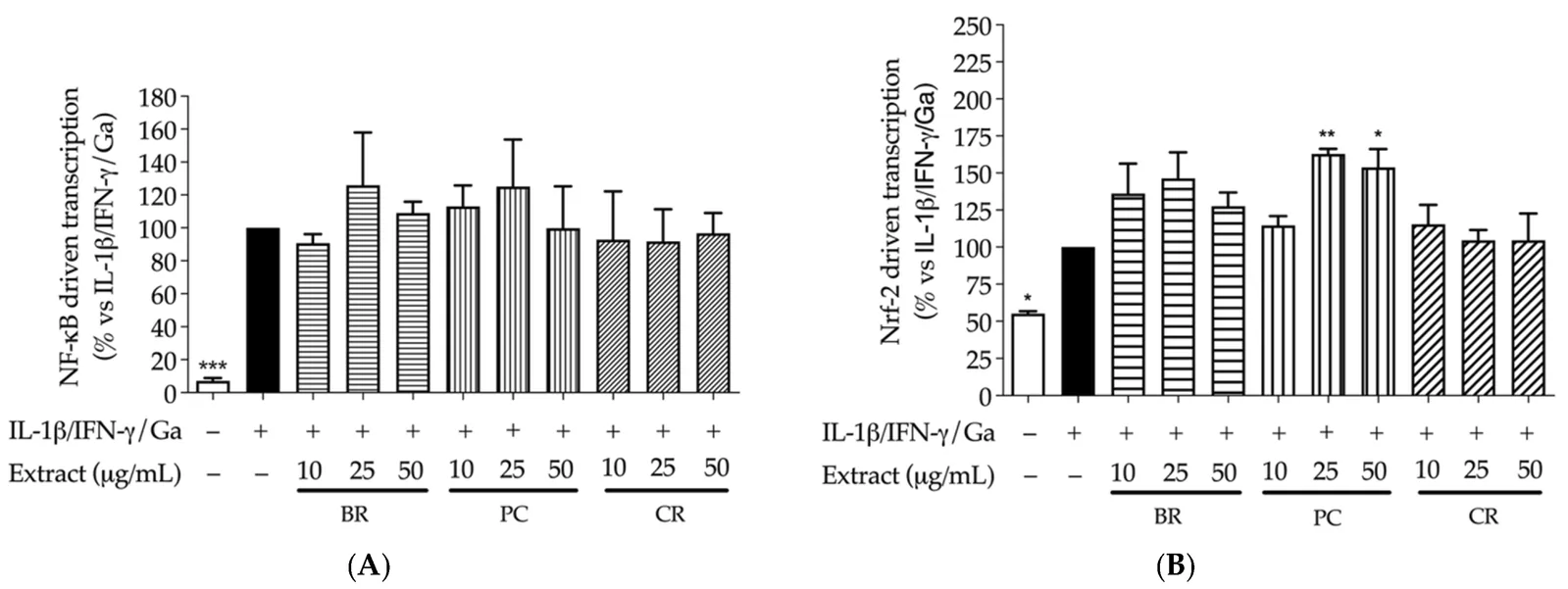
Effect of Brassicaceae sprout extracts on NF-κB (**A**) and Nrf-2 (**B**) driven transcription in Caco-2 cells measured by luciferase assay. Cells were treated with sprout extracts and pro-inflammatory cocktail (IL-1β, IFN-γ, Ga) for 6 h. BR (broccoli), PC (pak-choi), CR (red cabbage) correspond to horizontal, vertical, and diagonal lines, respectively; unstimulated control and pro-inflammatory stimulus correspond to white and black column, respectively. Apigenin 20 µM was used as reference inhibitor of NF-κB (–99%), while resveratrol 20 µM was used as a reference inducer of Nrf-2 (+432%). Data from *n* = 3 independent experiments were reported as mean luciferase activity (±SEM) vs. normalized stimulus (100%). * *p* < 0.05, ** *p* < 0.01, *** *p* < 0.001 vs. stimulus (one-way ANOVA).

MCP-1 is a well-known NF-κB-dependent mediator [31], and sulfur compounds are known to modulate NF-κB and Nrf-2 under inflammatory and oxidative conditions. Thus, the roles of these transcription factors in the observed biological activity were investigated. None of the extracts significantly inhibited NF-κB-driven transcription under the experimental conditions used (Figure 2A). PC significantly enhanced the Nrf-2 driven transcription at 25 and 50 µg/mL, whereas BR and CR showed no significant effect (Figure 2B). Again, the higher amount of sulfur compounds, specifically those present at higher concentrations in CR, was not correlated with Nrf-2 activation.

**Figure 3:**
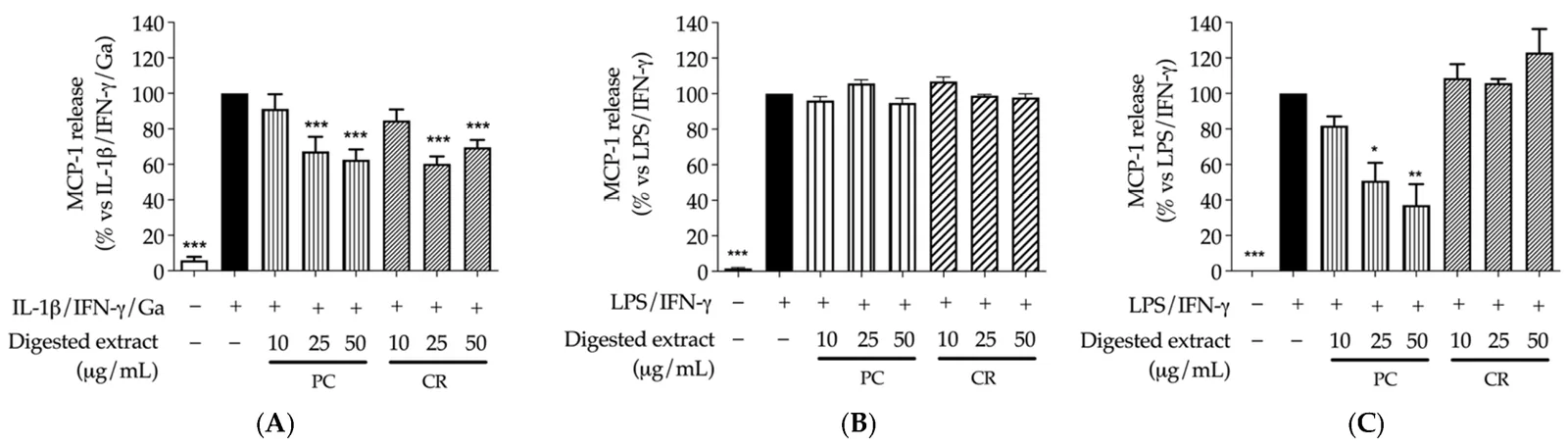
Effect of Brassicaceae sprout extracts (PC, CR) on the MCP-1 release measured by ELISA. Caco-2 cells were treated with digested sprout extracts and pro-inflammatory cocktail (IL-1β, IFN-γ, Ga) for 24 h (**A**). The co-culture of Caco-2 and THP-1 (Transwell^®^ plate) was treated with sprouts extracts and LPS/IFN-γ for 24 h (**B**). THP-1 cells were treated with sprout extracts and LPS/IFN-γ for 24 h; IC_50_ (PC) of 33.85 µg/mL (CI 95% from 20.84 to 54.98) was measured (**C**). PC (pak-choi) and CR (red cabbage) correspond to vertical and diagonal lines, respectively; unstimulated control and pro-inflammatory stimulus correspond to white and black columns, respectively. Apigenin 20 µM was used as a reference inhibitor in both Caco-2 (–73%) and THP-1 (–37%) models. Data from *n* = 3 independent experiments were reported as mean of release (±SEM) vs. normalized stimulus (100%). * *p* < 0.05, ** *p* < 0.01, *** *p* < 0.001 vs. stimulus (one-way ANOVA).

Total polyphenols were quantified as additional functional compounds potentially involved in the biological activity. In parallel, experiments were conducted to assess the chemical and biological stability of the most promising Brassicaceae sprout extracts, PC and CR, after theoretical oral consumption. For this purpose, samples were processed by simulated gastrointestinal digestion. Broccoli sprout extract contained 3.64 ± 0.31% *w*/*w* (36.4 mg GA eq./g of extract) of total polyphenols; substantial amounts were measured in the other two extracts (approximately 3.5% and 4.5%, respectively), which were only slightly reduced in PC and slightly increased in CR after digestion (Table 4). The total anthocyanins and flavonols content was also measured, accounting for a relevant fraction of total polyphenols. The amount of anthocyanins aligns with the established trend observed for CR, which exhibited a substantial increase after digestion. Total flavonols were relatively less affected in CR with respect to PC after digestion. Similarly, both extracts demonstrated radical scavenging activity in the ORAC and DPPH assays, with CR showing stronger activity in the DPPH assay (Table 5). These observations may be related to the higher polyphenol content of CR, particularly after digestion. Again, the inhibitory effect on MCP-1 was maintained (Figure 3A), further supporting the potential stability of the active components in the gastrointestinal environment.

Based on these results, digested sprout extracts were also investigated in differentiated Caco-2 cells grown on Transwell^®^ plates and co-cultured with activated THP-1 macrophages (LPS/IFN-γ) to explore the impact on epithelial permeability. Unexpectedly, the inhibitory effect on MCP-1 was lost in this experimental setting (Figure 3B). However, PC caused a concentration-dependent reduction in the same marker in activated THP-1 cells, separately cultivated, with an IC_50_ of 33.85 µg/mL (CI 95% from 20.84 to 54.98) (Figure 3C).

**Figure 4:**
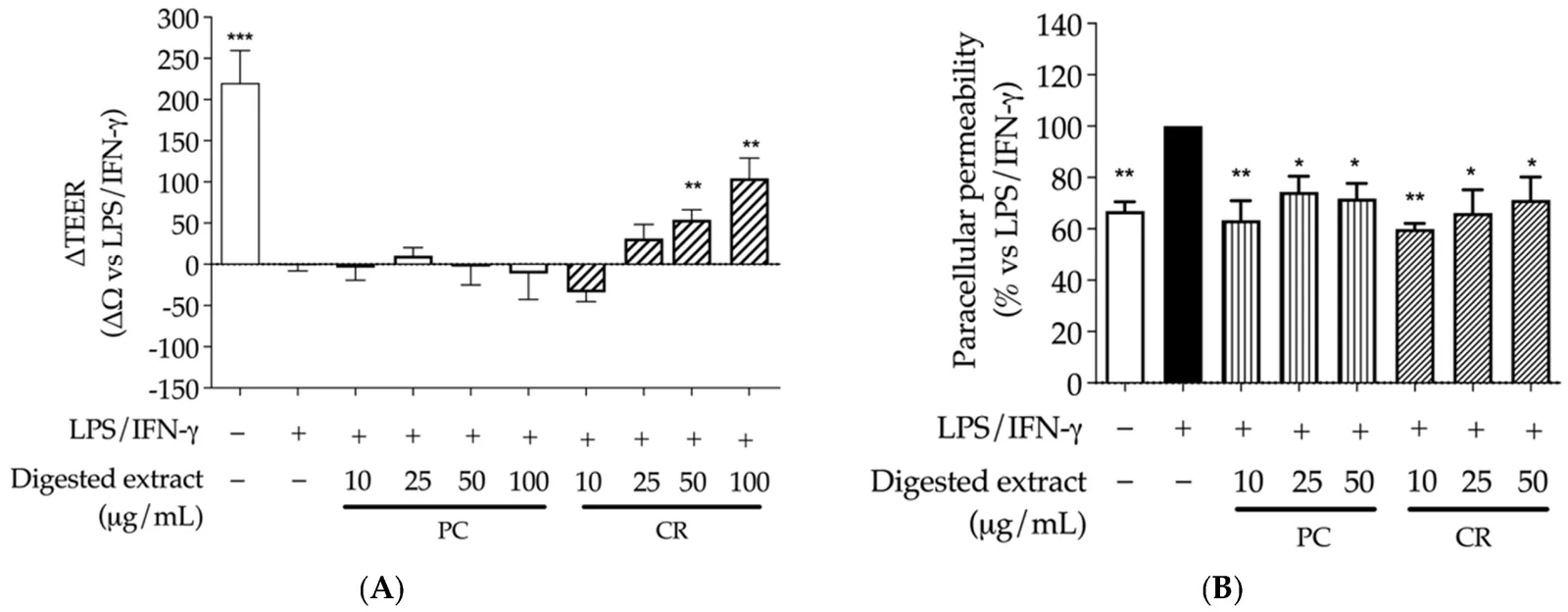
Effect of Brassicaceae sprout extracts (PC, CR) on intestinal epithelial permeability. The co-culture of Caco-2 and THP-1 (Transwell^®^ plate) was treated with sprout extracts and LPS/IFN-γ for 48 h. The variation in TEER (ΔΩ) (**A**) and the basolateral diffusion of Lucifer Yellow (LY, paracellular permeability) (**B**) were measured. PC (pak-choi) and CR (red cabbage) correspond to vertical and diagonal lines, respectively; unstimulated control and pro-inflammatory stimulus correspond to white and black columns, respectively. GFN 50 µM was used as a reference compound (ΔΩ = +60). Data from *n* = 3 independent experiments were reported as mean (±SEM) vs. normalized stimulus. * *p* < 0.05, ** *p* < 0.01, *** *p* < 0.001 vs. stimulus (one-way ANOVA).

The experiments related to epithelial permeability included the measurement of TEER values (epithelial integrity) and LY diffusion (paracellular permeability) at a later timepoint (48 h). PC was unable to recover TEER values, whereas CR caused a partial recovery in a concentration-dependent manner (10–100 µg/mL) (Figure 4A). The activity was comparable to that of GFN 50 µM (ΔΩ = +60) used as a reference compound in our previous work [24]. In the LY experiments, both extracts recovered the paracellular permeability at all concentrations tested (10-25-50 µg/mL) (Figure 4B). Thus, CR improved both electrical resistance and paracellular tracer permeability, whereas the effect of PC was detectable only in the LY assay.

These data prompted a wider investigation, aimed at describing the modulatory effects on inflammation and epithelial barrier function occurring at the transcriptional level in the co-culture model. GFN (50 µM) was included for comparison to better understand the role of sulfur compounds in the biological activity of PC and CR. For this purpose, 48 genes involved in cell damage, inflammation, oxidative stress, enterocyte differentiation, and epithelial barrier function were selected for a customized PCR array (the gene list with RefSeq numbers is reported in Table S2). The overall summary of regulated genes and fold changes is reported in Table S3, while genes showing nominally significant changes before FDR correction are reported in Table 6.

**Figure 5:**
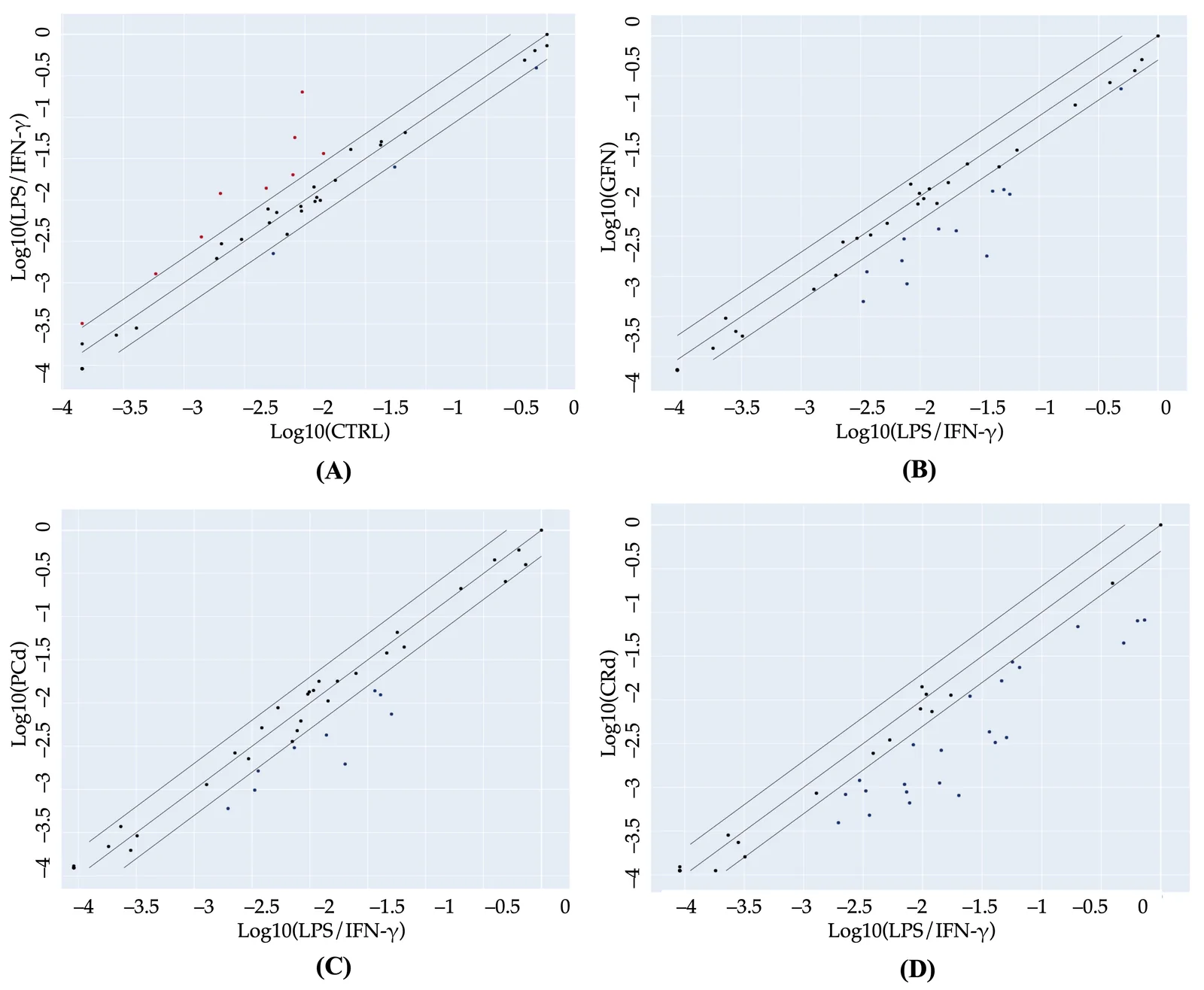
Scatter plots showing the expression profiles of inflammatory genes analyzed by PCR array in Caco-2. The Caco-2/THP-1 co-culture system (Transwell^®^ plate) was treated with sprout extracts and LPS/IFN-γ for 24 h. Scatter plots comparing the unstimulated control (**A**), GFN 50 µM (**B**), digested PC (PCd) 50 µg/mL (**C**), and digested CR (CRd) 50 µg/mL (**D**) with the LPS/IFN-γ-stimulated condition are shown. The central diagonal line represents unchanged gene expression (black dots), whereas the outer diagonal lines indicate the selected fold regulation threshold. Data points located beyond the outer lines in the upper-left and lower-right quadrants represent genes that were up-regulated (red dots) or down-regulated (blue dots), respectively, by more than the selected fold regulation threshold in the y-axis group compared with the x-axis group. Scatter plots were automatically generated using the GeneGlobe data analysis web portal (QIAGEN).

The modulation of a few genes was nominally significant (*p*-values), while none of the genes remained significantly modulated after multiple testing corrections (*q*-values). However, the pro-inflammatory condition (LPS/IFN-γ) was correlated with over-expression of chemokines (CCL20, CXCL-10), pro-inflammatory enzymes (TGM2, NOS2), tight-junction-related proteins (OCLN, CLDN1, ICAM1), and transcription factors (NFKBIA, STAT3); several antioxidant enzymes were instead down-regulated (CAT, NQO1) (Figure 5A). The scatter plots reported in Figure 5 showed that some genes were down-regulated following treatment with GFN (Figure 5B) and with digested sprout extracts tested at a concentration of 50 µg/mL (Figure 5C,D). Some genes remained unchanged, and none was over-expressed. Several genes involved in epithelial barrier function (TGM2, CLDN1, OCLN) were regulated in the opposite direction to that observed under the pro-inflammatory condition by GFN and the sprout extracts; another group of genes involved in cytoskeleton and barrier remodeling (HDAC2, HMOX1, RHOA, F2RL1) was down-regulated by GFN and the sprout extracts regardless of the inflammatory condition. These observations suggested an interesting degree of overlap among the glucosinolate and the sprout samples. Other genes related to inflammatory transcription factors were down-regulated only by CR or PC (STAT3, NFKBIA), regardless the role of GFN, which reflected the contribution of other functional compounds such as polyphenols in transcriptional mechanisms. However, although inflammatory transcription factors were negatively modulated, the effect of sprout extracts was not reflected in the modulation of chemokines.

The overall summary of regulated genes and fold changes is reported in Table S3, while significantly regulated genes and fold changes are described in Table 6.

## 4. Discussion

The present study compared the hydroalcoholic extracts of *Brassica* sprouts obtained from broccoli (BR), red cabbage (CR), and pak-choi (PC) in terms of bioactive compounds and anti-inflammatory activity. The framework of investigation focused on chronic gut inflammation, including celiac disease, which was selected due to the natural absence of gluten in *Brassica* sprouts and the lack of comparative studies in this context. Moreover, our group has previously suggested that GFN and SFN may contribute to gut barrier defense through diverse biological mechanisms [24]. The choice to investigate sprout extracts was driven by the numerous factors that influence the bioavailability of sulfur compounds during the direct consumption of these vegetables. The content of bioactive molecules is known to be influenced by harvesting, growth stage, food processing, cooking, and food matrix [9,10]. Thus, standardized sprout extracts may represent a more valuable source of sulfur compounds for nutraceutical applications compared to whole food products.

The first screening showed that GFN and SFN were less represented in BR, in respect to CR and PC (Table 3). The inclusion of BR as a comparator was intended to contextualize the phytochemical composition and biological activity of the less extensively studied CR and PC sprouts against one of the most widely investigated *Brassica* sprout matrices. The result was surprising, given the high level of attention dedicated to broccoli sprouts by researchers and nutraceutical companies; however, other studies have shown that *Brassica* sprouts other than broccoli may represent an equal or superior source of glucosinolates [13,14]. The present findings should be interpreted as specific to the plant material and extraction batch investigated here and should not be generalized to broccoli sprouts as a whole. We based our comparative work on the hydroalcoholic extraction from food matrixes (50:50 water:ethanol, room temperature) as a standard technique to obtain stable extracts enriched in middle polar to polar compounds. The extraction method was rationally selected from a previous work [25], in which the inhibitory role of ethanol >40% on myrosinase has been described. However, the same authors suggested that optimal extraction of glucosinolates has been obtained by applying higher volumes of ethanol and higher temperatures with respect to ours (up to 80% ethanol, at 60 °C). In line with this statement, other authors analyzed the glucosinolates using ethanol/water or methanol/water solvents with ratio 70:30, and higher temperatures [32,33]. Thus, a residual activity of myrosinase after extraction cannot be excluded in our samples.

It has also been reported that genetic factors may preferentially affect the content of aliphatic glucosinolates, such as GFN [34]. Another limitation of our study is the lack of characterization of glucosinolates other than GFN; nevertheless, the same authors quantified the total amount of glucosinolates from broccoli sprouts (0.5 g/100 g), showing values lower than those measured in our CR sample.

Cultivation conditions are another recognized variable: to the best of our knowledge, few comparative studies have been conducted to address the impact of cultivation on GFN content; however, the role of germination stage and sulphate supply is well-known [35].

Broccoli sprouts also exhibited a slightly lower inhibitory activity on MCP-1 release induced by a gliadin-containing inflammatory cocktail in Caco-2 cells (Figure 1). For this reason, PC and CR were selected for further analysis, revealing that the inhibitory activity was maintained after simulated gastrointestinal digestion (Figure 3A).

Since sulfur compounds are well-known inhibitors of NF-κB and inducers of Nrf-2, several experiments were dedicated to correlate their mechanism of action with their amount in extracts. Unexpectedly, none of the *Brassica* sprout extracts impaired the NF-κB-driven transcription (Figure 2A), whereas only PC significantly enhanced the Nrf-2 activation (Figure 2B). Taken together, these findings indicate that the inhibition of MCP-1 release by the three extracts cannot be primarily attributed to suppression of NF-κB-driven transcription. Other transcriptional, post-transcriptional, or secretion-related mechanisms should therefore be considered.

The initial body of data allowed us to correlate the presence of sulfur compounds to the biological effects of PC and CR. Consistent with the profile of the sprout extracts selected for the present study, our previous findings demonstrated that SFN exerts a stronger effect on MCP-1 release (IC_50_ = 7.81 µM) [24]. Furthermore, the inhibitory mechanism of SFN on MCP-1 release has previously been attributed to Nrf-2 activation in another cellular context [36]. However, from a quantitative perspective, the biological findings did not show a straightforward relationship between the abundance of the quantified sulfur compounds and the activity of the extracts. PC produced the strongest inhibition of MCP-1 release and was the only extract able to activate Nrf-2 (Figure 3C), despite containing markedly lower amounts of GFN and SFN than CR. Conversely, CR, which contained the highest levels of both compounds, did not activate Nrf-2 but showed the clearest effects on epithelial barrier readouts. These divergent profiles indicate that different phytochemical components, or interactions among them, may contribute to distinct biological effects. For this reason, total polyphenols were also measured, assuming that the polyphenolic fraction was relevant and stable after a simulated digestion process, especially in CR (Table 4). Our results were comparable with those of other published studies [4,37], in which amounts ranging from 90 to 300 mg GAE equivalents/100 g fresh weight of total polyphenols were measured in *Brassica* spp. extracts: considering the extraction yield (Table 2), PC and CR were close to 70 and 90 mg GAE equivalents/100 g of fresh weight, respectively. Considering that SFN and GFN are devoid of scavenging properties [24,38], the antioxidant capacity measured by DPPH and ORAC reported in Table 5 may reflect, at least in part, the contribution of the phenolic fraction; however, these assays assess chemical radical-scavenging capacity and do not directly reflect intracellular antioxidant activity or epithelial barrier protection. A limitation of our study is the lack of a comprehensive characterization of the phenolic composition; however, this data has been extensively reported by a few articles, in which the presence of flavonols (mostly quercetin and kaempferol derivatives), anthocyanins, hydroxycinnamic acid (such as ferulic and sinapic acid), and hydroxybenzoic acid derivatives has been identified in *Brassica* sprouts [39,40,41]. The impact of a gastrointestinal simulated digestion was also described: in line with our data, the bioaccessibility of polyphenols was lower in broccoli sprouts than in red cabbage, with a major role for hydroxycinnamic acids [41].

We previously suggested that GFN should be considered not only as a precursor of bioactive ITCs, but also a bioactive molecule itself, particularly regarding its protective effects on epithelial integrity [24]. The same result was simultaneously supported by the experimental work of Zhu and colleagues [42], in which the protective effect of GFN (1 µM) on the intestinal barrier was partially attributed to redox pathways. In the co-culture experiments, CR showed protective effects on epithelial barrier integrity, as measured by TEER and Lucifer Yellow assays (25–100 µg/mL), whereas PC was unable to recover TEER values (Figure 4). The partial discrepancy between TEER and Lucifer Yellow results is not necessarily contradictory, because the two assays assess complementary features of epithelial barrier function. TEER predominantly reflects ionic conductance across the monolayer, whereas Lucifer Yellow diffusion measures the paracellular passage of a hydrophilic tracer. The activity of PC in the Lucifer Yellow assay, in the absence of TEER recovery, may therefore indicate a more selective or less extensive effect on paracellular permeability.

The PCR array provided preliminary transcriptional information that may help generate hypotheses regarding the effects of CR; however, it did not establish a direct molecular explanation for the functional barrier findings (Table S3). Moreover, none of the nominally reduced changes obtained by gene-by-gene analysis remained significant after multiple testing corrections across the 48 genes (see *q*-values in Table 6), thus limiting the relevance of mechanistic findings. Several genes, including CLDN1, were modulated by the sprout extracts and GFN, thus suggesting a certain degree of overlap with the reference sulfur compound. CR reduced CLDN1 transcript levels relative to the inflammatory condition, while improving both TEER and Lucifer Yellow permeability. Although this finding may reflect a partial reversal of the transcriptional response induced by LPS/IFN-γ, the functional significance of CLDN1 down-regulation remains unclear. The reduction in STAT3 transcripts by CR may be consistent with the SFN content and the modulation of inflammatory signaling but deserves further mechanistic investigation. Nevertheless, the activation of STAT3 and the perturbation of CLDN1 expression have been documented in inflammatory-based bowel disorders, including celiac disease [15,16]. Accordingly, Chen and colleagues suggested that SFN (10 mg/kg) ameliorated DSS-induced colitis by modulating the STAT3 pathway in an Nrf2-dependent manner [43]. Thus, the overall data are consistent with the hypothesis that several phytochemical components of CR, including polyphenols and sulfur compounds, might be responsible for gut barrier protective mechanisms potentially relevant to disorders associated with leaky gut; however, the present study does not allow the specific contribution of the different compound classes or any potential interactions between them to be established. Although the role of CR in gut inflammation remains largely unexplored, a recent dietary intervention study investigated the tolerability of consuming 30 g/day of red cabbage sprouts, attributing beneficial effects on inflammatory gastrointestinal symptoms to this amount [44]. The concentration of extracts used in our study may plausibly reflect realistic exposure at a gut level. Based on the phytochemical characterization, the treatment with 100 µg/mL of sprout extracts corresponds to approximately 0.55 µM SFN and 0.02 µM GFN for PC, and 5.37 µM SFN and 5.06 µM GFN for CR. According to the extraction yield (close to 2%), 100 µg/mL of extract should correspond to 5 mg of fresh sprouts, which is far below a common consumption of a sprouts serving (30–80 g). From a pharmacokinetic point of view, human studies have reported micromolar plasma concentrations of ITCs following consumption of 40 g of broccoli sprouts or an equivalent preparation to 200 µM of ITCs [45,46,47].

A limitation of the present study is that only one cultivation and extraction batch was investigated for each *Brassica* sprout species. Therefore, the observed phytochemical composition and biological activity may have been influenced by batch-specific or cultivation-related factors, and further studies including different cultivars and independent production batches are needed to confirm the reproducibility of these findings. Moreover, the present study was conducted exclusively using in vitro models and did not address the intestinal bioavailability or in vivo efficacy of the investigated extracts. Although the Caco-2 and Caco-2/THP-1 models provide complementary approaches to investigate inflammatory responses and epithelial barrier function, they represent simplified in vitro systems and cannot recapitulate the complex pathophysiology of celiac disease or inflammatory bowel disease. In addition, although several major bioactive compounds were quantified, the phytochemical characterization was not comprehensive. Therefore, the present findings should be considered preliminary, and further studies integrating comprehensive phytochemical profiling, bioavailability assessment, and in vivo investigations are demanded to substantiate the potential nutraceutical relevance of standardized *Brassica* sprout extracts.

## Figures and Tables

**Table 1:** Summary of 48 genes included in customized PCR array with their reference cluster. Housekeeping genes and PCR quality control are also described.

Cluster	Symbol
Cell damage (alarmins) and inflammation	1. IL15
	2. HMGB1
	3. IL7
	4. F2RL1
	5. IL33
	6. TGM2
	7. MMP9
	8. CCL2
	9. MMP3
	10. EGFR
	11. CXCL10
	12. TSLP
	13. CXCL8
	14. NOS2
	15. CXCL11
	16. CXCR3
	17. CCL20
	18. DEFB4A
Tight junctions and cytoskeleton regulators	19. TJP1
	20. TJP2
	21. TJP3
	21. OCLN
	22. ICAM1
	23. RHOA
	24. CLDN1
	25. ALPI
	26. MYLK
Oxidative stress	27. CAT
	28. SOD1
	29. HMOX1
	30. NQO1
Enterocyte differentiation	31. SLC5A1
	32. SERPINA1
	33. APOA1
	34. APOC3
Transcription factors and gene regulators	35. HDAC1
	36. HDAC2
	37. PPARG
	38. STAT1
	39. NFKBIA
	40. STAT3
	41. PPARA
Housekeeping genes	42. RPLP0
	43. HPRT1
	44. GAPDH
PCR controls	45. RTC
	46. PPC
	47. HGDC

**Table 2:** Yields of extraction of fresh Brassicaceae sprouts.

Brassicaceae Sprouts	(% *w*/*w* Fresh Material)
BR	1.81
PC	1.73
CR	2.07

BR Broccoli Sprouts; PC, Pak-Choi Sprouts; CR, Red Cabbage Sprouts; *w*, Weight.

**Table 3:** LC-MS quantification of SFN and GFN in Brassicaceae sprout extracts.

Brassicaceae Sprouts Extracts	SFN (mg/g of Extract ± SD)	GFN (mg/g of Extract ± SD)
BR	n.d.	n.d.
PC	0.973 ± 0.055	0.067 ± 0.006
CR	9.517 ± 0.661	22.133 ± 0.212

BR, broccoli sprouts; PC, pak-choi sprouts; CR, red cabbage sprouts; SFN, sulforaphane; GFN, glucoraphanin; n.d., not detectable (for LOD and LOQ values, refer to Methods, Section 2.9).

**Table 4:** Total polyphenols, anthocyanins, and flavonols content before and after digestion (CR and PC).

Brassicaceae Sprouts Extracts	Before Digestion	After Digestion
Total polyphenols (mg GA eq./g of extract ± SD)		
PC	35.20 ± 2.56	31.29 ± 0.88 *
CR	45.74 ± 5.62	49.79 ± 0.44 ^ns^
Total anthocyanins (mg Cyanidin eq./g of extract ± SD)		
PC	2.32 ± 0.99	01 ± 0.42 **
CR	4.16 ± 0.35	9.30 ± 0.26 ^###^
Total flavonols (mg Quercetin eq./g of extract ± SD)		
PC	10.05 ± 0.15	0.72 ± 0.03 ***
CR	3.32 ± 0.12	0.58 ± 0.01 ^###^

PC, pak-choi sprout extract; CR, red cabbage sprout extract; GA eq., gallic acid equivalents *, **, *** *p*-value vs. PC; ^###^ *p*-value vs. CR; ^ns^, not significant (unpaired *t* test).

**Table 5:** ORAC and DPPH after digestion (CR and PC).

Brassicaceae Sprouts Extracts	ORAC (µM Trolox eq. ± SD)	DPPH (µM Trolox eq. ± SD)
PCd	26.42 ± 13.89	66.46 ± 5.00
CRd	26.79 ± 13.58	122.47 ± 9.00 ***

PCd, digested pak-choi sprout extract; CRd, digested red cabbage sprout extract; *** *p*-value vs. PCd (unpaired *t* test).

**Table 6:** Genes showing nominally significant expression changes before FDR correction.

**CTRL vs. LPS/IFN-γ**			
**Gene symbol**	**Fold regulation**	***p*-Value**	***q*-Value**
STAT3	2.9	0.028380	0.624360
CCL20	21.20	0.001749	0.076956
**LPS/IFN-γ vs. GFN**			
**Gene symbol**	**Fold regulation**	***p*** **-Value**	***q*** **-Value**
CLDN1	–20.31	0.037115	0.526213
**LPS/IFN-γ vs. CRd**			
**Gene symbol**	**Fold regulation**	***p*-Value**	***q*-Value**
CLDN1	–8.44	0.043282	0.474053
STAT3	–12.37	0.038667	0.474053

GFN, Glucoraphanin; CRd, Digested Red Cabbage Sprouts Extract.

## Data Availability

The original contributions presented in this study are included in the article/Supplementary Materials. Further inquiries can be directed to the corresponding author.

## Supplementary Materials

The following supporting information can be downloaded at: https://www.mdpi.com/article/10.3390/foods15193494/s1, Figure S1: Cell viability measured by MTT test; Figure S2: Effect of Brassicaceae sprouts extracts (BR, PC, CR) on the release of CXCL-10 (A) and IL-8 (B) by CaCo-2 cells measured by ELISA assay; Table S1: LC-MS quantification of SFN and GFN in Brassicaceae sprouts after freeze-drying; Table S2: Genes included in the customized PCR RT^2^ Profiler^TM^ (QIAGEN, Hilden, Germany) with respective RefSeq number; Table S3. Regulated genes with fold changes and *p*-Values.

## Abbreviations

The following abbreviations are used in this manuscript:

BR	Broccoli sprouts
PC	Pak-choi sprouts
CR	Red cabbage sprouts
SFN	Sulforaphane
GFN	Glucoraphanin
IL-1β	Interleukin-1β
IFN-γ	Interferon-γ
LPS	Lipopolysaccharide
TEER	Trans-epithelial electrical resistance
LY	Lucifer Yellow
GA eq.	Gallic acid equivalents
STAT3	Signal Transducer and Activator of Transcription 3
CLDN1	Claudin 1
NF-κB	Nuclear factor kappa-light-chain-enhancer of activated B cells
ITCs	Isothiocyanates
IL-8	Interleukin 8
CXCL-10	Chemokine (C-X-C motif) Ligand 10
MCP-1	Monocyte Chemoattractant Protein 1
Nrf-2	Nuclear redox factor 2

## References

[1] Tse G., Eslick G.D. (2014). Cruciferous vegetables and risk of colorectal neoplasms: A systematic review and meta-analysis. Nutr. Cancer.

[2] Connolly E.L., Liu A.H., Radavelli-Bagatini S., Shafaei A., Boyce M.C., Wood L.G., McCahon L., Koch H., Sim M., Hill C.R. (2024). Cruciferous vegetables lower blood pressure in adults with mildly elevated blood pressure in a randomized, controlled, crossover trial: The VEgetableS for vaScular hEaLth (VESSEL) study. BMC Med..

[3] Fowke J.H., Chung F.L., Jin F., Qi D., Cai Q., Conaway C., Cheng J.R., Shu X.O., Gao Y.T., Zheng W. (2003). Urinary isothiocyanate levels, brassica, and human breast cancer. Cancer Res..

[4] Sabin O., Pop R.M., Bocsan I.C., Chedea V.S., Ranga F., Grozav A., Levai A.M., Buzoianu A.D. (2024). The Anti-Inflammatory, Analgesic, and Antioxidant Effects of Polyphenols from Brassica oleracea var. capitata Extract on Induced Inflammation in Rodents. Molecules.

[5] Choi H., Kim H., Han S., Park H.W., Ha I.J., Kim J.S., Lee S.G. (2023). Antioxidant and Anti-Inflammatory Activities of High-Glucosinolate-Synthesis Lines of Brassica rapa. Antioxidants.

[6] Novio S., Cartea M.E., Soengas P., Freire-Garabal M., Nunez-Iglesias M.J. (2016). Effects of Brassicaceae Isothiocyanates on Prostate Cancer. Molecules.

[7] Bessler H., Djaldetti M. (2018). Broccoli and human health: Immunomodulatory effect of sulforaphane in a model of colon cancer. Int. J. Food Sci. Nutr..

[8] Sturm C., Wagner A.E. (2017). Brassica-Derived Plant Bioactives as Modulators of Chemopreventive and Inflammatory Signaling Pathways. Int. J. Mol. Sci..

[9] Yagishita Y., Fahey J.W., Dinkova-Kostova A.T., Kensler T.W. (2019). Broccoli or Sulforaphane: Is It the Source or Dose That Matters?. Molecules.

[10] Kushad M.M., Brown A.F., Kurilich A.C., Juvik J.A., Klein B.P., Wallig M.A., Jeffery E.H. (1999). Variation of glucosinolates in vegetable crops of Brassica oleracea. J. Agric. Food Chem..

[11] West L.G., Meyer K.A., Balch B.A., Rossi F.J., Schultz M.R., Haas G.W. (2004). Glucoraphanin and 4-hydroxyglucobrassicin contents in seeds of 59 cultivars of broccoli, raab, kohlrabi, radish, cauliflower, brussels sprouts, kale, and cabbage. J. Agric. Food Chem..

[12] Bhandari S.R., Jo J.S., Lee J.G. (2015). Comparison of Glucosinolate Profiles in Different Tissues of Nine Brassica Crops. Molecules.

[13] Yamaguchi Y., Sugiki M., Shimizu M., Ogawa K., Kumagai H. (2024). Comparative analysis of isothiocyanates in eight cruciferous vegetables and evaluation of the hepatoprotective effects of 4-(methylsulfinyl)-3-butenyl isothiocyanate (sulforaphene) from daikon radish (*Raphanus sativus* L.) sprouts. Food Funct..

[14] De Nicola G.R., Bagatta M., Pagnotta E., Angelino D., Gennari L., Ninfali P., Rollin P., Iori R. (2013). Comparison of bioactive phytochemical content and release of isothiocyanates in selected brassica sprouts. Food Chem..

[15] Barmeyer C., Fromm M., Schulzke J.D. (2017). Active and passive involvement of claudins in the pathophysiology of intestinal inflammatory diseases. Pflug. Arch..

[16] Zhu L., Han J., Li L., Wang Y., Li Y., Zhang S. (2019). Claudin Family Participates in the Pathogenesis of Inflammatory Bowel Diseases and Colitis-Associated Colorectal Cancer. Front. Immunol..

[17] Di Vincenzo F., Del Gaudio A., Petito V., Lopetuso L.R., Scaldaferri F. (2024). Gut microbiota, intestinal permeability, and systemic inflammation: A narrative review. Intern. Emerg. Med..

[18] Lipska P., Lukanko K., Sobczak J., Lazarchuk I., Duda-Madej A. (2026). From Dysbiosis to Inflammation: Gut Microbiota and Oxidative Stress in Atopic Dermatitis. Antioxidants.

[19] Yanaka A., Fahey J.W., Fukumoto A., Nakayama M., Inoue S., Zhang S., Tauchi M., Suzuki H., Hyodo I., Yamamoto M. (2009). Dietary sulforaphane-rich broccoli sprouts reduce colonization and attenuate gastritis in Helicobacter pylori-infected mice and humans. Cancer Prev. Res..

[20] Holman J., Hurd M., Moses P.L., Mawe G.M., Zhang T., Ishaq S.L., Li Y. (2023). Interplay of broccoli/broccoli sprout bioactives with gut microbiota in reducing inflammation in inflammatory bowel diseases. J. Nutr. Biochem..

[21] Wei L.Y., Zhang J.K., Zheng L., Chen Y. (2022). The functional role of sulforaphane in intestinal inflammation: A review. Food Funct..

[22] Garcia-Perez P., Tomas M., Giuberti G., Capanoglu E., Callegari M.L., Lucini L., Patrone V. (2025). Brassica microgreens shape gut microbiota and functional metabolite profiles in a species-related manner: A multi-omics approach following in vitro gastrointestinal digestion and large intestine fermentation. Microbiol. Res..

[23] Kaulmann A., Planchon S., Renaut J., Schneider Y.J., Hoffmann L., Bohn T. (2016). Proteomic response of inflammatory stimulated intestinal epithelial cells to in vitro digested plums and cabbages rich in carotenoids and polyphenols. Food Funct..

[24] Sonzogni E., Martinelli G., Fumagalli M., Maranta N., Pozzoli C., Bani C., Marrari L.A., Di Lorenzo C., Sangiovanni E., Dell’Agli M. (2024). In Vitro Insights into the Dietary Role of Glucoraphanin and Its Metabolite Sulforaphane in Celiac Disease. Nutrients.

[25] Albe-Slabi S., Tollitte A., Burgel P.O.F., Beau L., Galet O., Mutelet F., Kapel R. (2025). Rational hydroalcoholic extraction strategy for the recovery of intact glucosinolates from rapeseed cold-pressed cake. Appl. Food Res..

[26] Lund M.E., To J., O’Brien B.A., Donnelly S. (2016). The choice of phorbol 12-myristate 13-acetate differentiation protocol influences the response of THP-1 macrophages to a pro-inflammatory stimulus. J. Immunol. Methods.

[27] Pozzoli C., Martinelli G., Fumagalli M., Di Lorenzo C., Maranta N., Colombo L., Piazza S., Dell’Agli M., Sangiovanni E. (2024). Castanea sativa Mill. By-Products: Investigation of Potential Anti-Inflammatory Effects in Human Intestinal Epithelial Cells. Molecules.

[28] O’Connell D.J., Kolde R., Sooknah M., Graham D.B., Sundberg T.B., Latorre I., Mikkelsen T.S., Xavier R.J. (2016). Simultaneous Pathway Activity Inference and Gene Expression Analysis Using RNA Sequencing. Cell Syst..

[29] Martinelli G., Fumagalli M., Pozzoli C., Nicotra G., Vicentini S.F., Maranta N., Sangiovanni E., Dell’Agli M., Piazza S. (2023). Exploring In Vitro the Combination of *Cistus* × *incanus* L. and *Castanea sativa* Mill. Extracts as Food Supplement Ingredients against H. pylori Infection. Foods.

[30] Sangiovanni E., Di Lorenzo C., Colombo E., Colombo F., Fumagalli M., Frigerio G., Restani P., Dell’Agli M. (2015). The effect of in vitro gastrointestinal digestion on the anti-inflammatory activity of *Vitis vinifera* L. leaves. Food Funct..

[31] Akhter N., Wilson A., Thomas R., Al-Rashed F., Kochumon S., Al-Roub A., Arefanian H., Al-Madhoun A., Al-Mulla F., Ahmad R. (2021). ROS/TNF-alpha Crosstalk Triggers the Expression of IL-8 and MCP-1 in Human Monocytic THP-1 Cells via the NF-kappaB and ERK1/2 Mediated Signaling. Int. J. Mol. Sci..

[32] Triska J., Balik J., Houska M., Novotna P., Magner M., Vrchotova N., Hic P., Jilek L., Thorova K., Snurkovic P. (2021). Factors Influencing Sulforaphane Content in Broccoli Sprouts and Subsequent Sulforaphane Extraction. Foods.

[33] De Nicola G.R., Nyegue M., Montaut S., Iori R., Menut C., Tatibouet A., Rollin P., Ndoye C., Zollo P.H. (2012). Profile and quantification of glucosinolates in Pentadiplandra brazzeana Baillon. Phytochemistry.

[34] Pérez-Balibrea S., Moreno D.A., García-Viguera C. (2011). Genotypic effects on the phytochemical quality of seeds and sprouts from commercial broccoli cultivars. Food Chem..

[35] Perez-Balibrea S., Moreno D.A., Garcia-Viguera C. (2010). Glucosinolates in broccoli sprouts (Brassica oleracea var. italica) as conditioned by sulphate supply during germination. J. Food Sci..

[36] Starrett W., Blake D.J. (2011). Sulforaphane inhibits de novo synthesis of IL-8 and MCP-1 in human epithelial cells generated by cigarette smoke extract. J. Immunotoxicol..

[37] Natella F., Maldini M., Nardini M., Azzini E., Foddai M.S., Giusti A.M., Baima S., Morelli G., Scaccini C. (2016). Improvement of the nutraceutical quality of broccoli sprouts by elicitation. Food Chem..

[38] Fahey J.W., Talalay P. (1999). Antioxidant functions of sulforaphane: A potent inducer of Phase II detoxication enzymes. Food Chem. Toxicol..

[39] Sun J., Xiao Z., Lin L.Z., Lester G.E., Wang Q., Harnly J.M., Chen P. (2013). Profiling polyphenols in five Brassica species microgreens by UHPLC-PDA-ESI/HRMS(n.). J. Agric. Food Chem..

[40] Bajkacz S., Ligor M., Baranowska I., Buszewski B. (2021). Separation and Determination of Chemopreventive Phytochemicals of Flavonoids from Brassicaceae Plants. Molecules.

[41] Abellan A., Dominguez-Perles R., Garcia-Viguera C., Moreno D.A. (2021). In Vitro Evidence on Bioaccessibility of Flavonols and Cinnamoyl Derivatives of Cruciferous Sprouts. Nutrients.

[42] Zhu W., Cremonini E., Mastaloudis A., Oteiza P.I. (2024). Glucoraphanin and sulforaphane mitigate TNFalpha-induced Caco-2 monolayers permeabilization and inflammation. Redox Biol..

[43] Chen M., Yu G., Wu W., Lei P., Zhang X., Ren J., Jiang X., Yang M., He C. (2026). Sulforaphane attenuates DSS-induced ulcerative colitis via the Nrf2/STAT3 signaling pathway and gut microbiota modulation. Food Funct..

[44] Lee S.Y., Michell K.A., Butler M.M., Smith B.T., Woolf E.K., Holmes S.C., Grabos L.E., Vazquez A.R., Isweiri H., Bunning M. (2025). Feasibility and Tolerability of Daily Microgreen Consumption in Healthy Middle-Aged/Older Adults: A Randomized, Open-Label, Controlled Crossover Trial. Nutrients.

[45] Clarke J.D., Hsu A., Riedl K., Bella D., Schwartz S.J., Stevens J.F., Ho E. (2011). Bioavailability and inter-conversion of sulforaphane and erucin in human subjects consuming broccoli sprouts or broccoli supplement in a cross-over study design. Pharmacol. Res..

[46] Atwell L.L., Hsu A., Wong C.P., Stevens J.F., Bella D., Yu T.W., Pereira C.B., Lohr C.V., Christensen J.M., Dashwood R.H. (2015). Absorption and chemopreventive targets of sulforaphane in humans following consumption of broccoli sprouts or a myrosinase-treated broccoli sprout extract. Mol. Nutr. Food Res..

[47] Ye L., Dinkova-Kostova A.T., Wade K.L., Zhang Y., Shapiro T.A., Talalay P. (2002). Quantitative determination of dithiocarbamates in human plasma, serum, erythrocytes and urine: Pharmacokinetics of broccoli sprout isothiocyanates in humans. Clin. Chim. Acta.

