# Supplementary material for: A Comparative Study of Brassica Sprout Extracts on Intestinal Inflammation and Epithelial Barrier Integrity
Source: Foods. 2026 Sep 29;15(19):3494. doi: 10.3390/foods15193494 (PMC13664419; doi:10.3390/foods15193494)
Supplement: Supplementary file 1 [file foods-15-03494-s001.zip › foods-4560806-supplementary.pdf]

Supplementary data

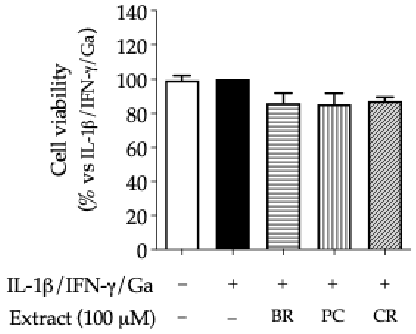

Figure S1. Cell viability measured by MTT test. CaCo-2 cells were treated with Brassicaceae sprouts extracts (BR, PC, CR) and pro-inflammatory cocktail (IL-1β, IFN-γ, Ga) for 24 h. Data from n=3 independent experiments were reported as mean of release (± SEM) vs. normalized stimulus (100 %). ***p<0.001 vs. stimulus (one-way ANOVA).

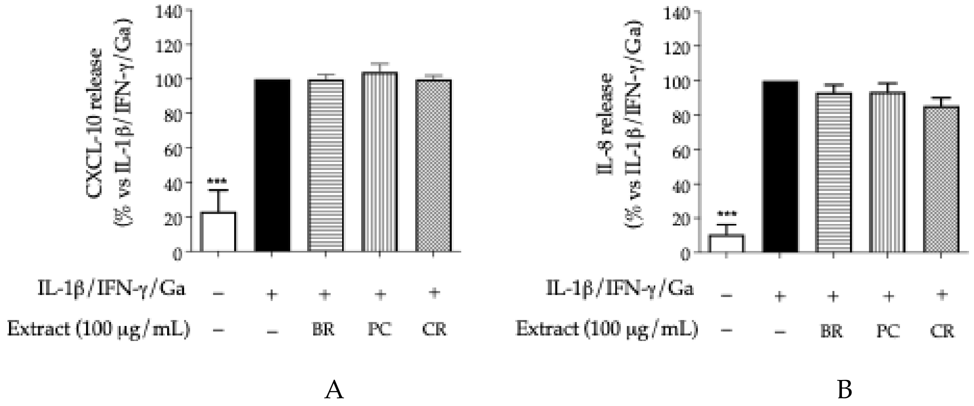

Figure S2. Effect of Brassicaceae sprouts extracts (BR, PC, CR) on the release of CXCL-10 (**A**) and IL-8 (**B**) by CaCo-2 cells measured by ELISA assay. Cells were treated with sprouts extracts and pro-inflammatory cocktail (IL-1β, IFN-γ, Ga) for 24 h. Apigenin 20 μM was used as reference inhibitor (-26 %) Data from n=3 independent experiments were reported as mean of release (± SEM) vs. normalized stimulus (100 %). ***p<0.001 vs. stimulus (one-way ANOVA).

Table S1. LC-MS quantification of SFN and GFN in Brassicaceae sprouts after freeze-drying

**Brassicaceae sprouts extracts**	**SFN** (mg/g of extract ± SD)	**GFN** (mg/g of extract ± SD)
CR	11.968 ± 3.024	20.457 ± 1.842
PC	1.033 ± 0.214	0.137 ± 0.021

Table S2. Genes included in the customized PCR RT^2 Profiler^{TM} (QIAGEN, Hilden, Germany) with respective RefSeq number.

Position	RefSeq Number	Symbol	Description
1	NM_000585	IL15	Interleukin 15
2	NM_004942	DEFB4A	Defensin, beta 4A
3	NM_014428	TJP3	Tight junction protein 3 (zona occludens 3)
4	NM_001752	CAT	Catalase

5	NM_002128	HMGB1	High mobility group box 1
6	NM_002538	OCLN	Occludin
7	NM_000880	IL7	Interleukin 7
8	NM_000343	SLC5A1	Solute carrier family 5 (sodium/glucose cotransporter), member 1
9	NM_005242	F2RL1	Coagulation factor II (thrombin) receptor-like 1
10	NM_000454	SOD1	Superoxide dismutase 1, soluble
11	NM_000201	ICAM1	Intercellular adhesion molecule 1
12	NM_001527	HDAC2	Histone deacetylase 2
13	NM_033439	IL33	Interleukin 33
14	NM_000295	SERPINA1	Serpin peptidase inhibitor, clade A (alpha-1 antiproteinase, antitrypsin), member 1
15	NM_002046	GAPDH	Glyceraldehyde-3-phosphate dehydrogenase
16	SA_00104	RTC	Reverse Transcription Control
17	NM_004613	TGM2	Transglutaminase 2 (C polypeptide, protein-glutamine-gamma-glutamyltransferase)
18	NM_004994	MMP9	Matrix metallopeptidase 9 (gelatinase B, 92kDa gelatinase, 92kDa type IV collagenase)
19	NM_002982	CCL2	Chemokine (C-C motif) ligand 2
20	NM_002133	HMOX1	Heme oxygenase (decycling) 1
21	NM_002422	MMP3	Matrix metallopeptidase 3 (stromelysin 1, progelatinase)
22	NM_001664	RHOA	Ras homolog gene family, member A
23	NM_005228	EGFR	Epidermal growth factor receptor
24	NM_015869	PPARG	Peroxisome proliferator-activated receptor gamma
25	NM_001565	CXCL10	Chemokine (C-X-C motif) ligand 10
26	NM_021101	CLDN1	Claudin 1
27	NM_007315	STAT1	Signal transducer and activator of transcription 1, 91kDa
28	NM_020529	NFKBIA	Nuclear factor of kappa light polypeptide gene enhancer in B-cells inhibitor, alpha
29	NM_033035	TSLP	Thymic stromal lymphopoietin
30	NM_175610	TJP1	Tight junction protein 1 (zona occludens 1)
31	NM_000194	HPRT1	Hypoxanthine phosphoribosyltransferase 1
32	nan	PPC	nan
33	NM_000584	CXCL8	Interleukin 8
34	NM_000625	NOS2	Nitric oxide synthase 2, inducible
35	NM_005409	CXCL11	Chemokine (C-X-C motif) ligand 11
36	NM_001504	CXCR3	Chemokine (C-X-C motif) receptor 3
37	NM_003150	STAT3	Signal transducer and activator of transcription 3 (acute-phase response factor)
38	NM_000039	APOA1	Apolipoprotein A-I
39	NM_000040	APOC3	Apolipoprotein C-III
40	NM_004964	HDAC1	Histone deacetylase 1
41	NM_005036	PPARA	Peroxisome proliferator-activated receptor alpha
42	NM_000903	NQO1	NAD(P)H dehydrogenase, quinone 1
43	NM_001631	ALPI	Alkaline phosphatase, intestinal
44	NM_004591	CCL20	Chemokine (C-C motif) ligand 20
45	NM_053025	MYLK	Myosin light chain kinase
46	NM_004817	TJP2	Tight junction protein 2 (zona occludens 2)

47	NM_001002	RPLP0	Ribosomal protein, large, P0
48	SA_00105	HGDC	Human Genomic DNA Contamination

Table S3. Regulated genes with fold changes and p-Values. The CaCo-2/THP-1 co-culture was treated with inflammatory stimuli (LPS/IFN-γ) and GFN (glucoraphanin) 50 µM, PCd (digested pak-choi) 50 µg/mL, and CRd (digested red cabbage) 50 µg/mL.

Ctrl vs. inflammatory stimulus			
**Position**	**Gene Symbol**	**Fold Regulation**	**p-Value**
6	OCLN	2.53	0.759488
11	ICAM1	5.99	0.095693
17	TGM2	2.57	0.162400
25	CXCL10	2.23	0.778128
26	CLDN1	2.56	0.062166
28	NFKBIA	6.90	0.069210
34	NOS2	2.19	0.289907
37	STAT3	2.90	0.028380
44	CCL20	21.20	0.001749
4	CAT	-2.21	0.269160
39	APOC3	-2.07	0.351906
42	NQO1	-2.43	0.935816

GFN versus inflammatory stimulus			
**Position**	**Gene Symbol**	**Fold Regulation**	**p-Value**
6	OCLN	-5.44	0.632808
9	F2RL1	-2.50	0.546345
12	HDAC2	-4.20	0.215269
17	TGM2	-3.14	0.299681
20	HMOX1	-6.87	0.126443
22	RHOA	-3.52	0.132259
26	CLDN1	-20.31	0.037115
27	STAT1	-4.50	0.364424
28	NFKBIA	-5.37	0.212919
30	TJP1	-9.63	0.305277
38	APOA1	-2.23	0.127964
41	PPARA	-3.68	0.115963

PCd versus inflammatory stimulus			
**Position**	**Gene Symbol**	**Fold Regulation**	**p-Value**
6	OCLN	-10.25	0.370570
8	SLC5A1	-3.26	0.712537
9	F2RL1	-2.41	0.407522
12	HDAC2	-6.81	0.093223
17	TGM2	-2.19	0.597166
20	HMOX1	-3.40	0.151277

22	RHOA	-3.28	0.231285
26	CLDN1	-2.63	0.148568
37	STAT3	-3.26	0.125080

CRd versus inflammatory stimulus

Position	Gene Symbol	Fold Regulation	p-Value
3	TJP3	-2.45	0.291734
4	CAT	-2.27	0.088931
5	HMGB1	-2.72	0.415080
6	OCLN	-24.89	0.161609
8	SLC5A1	-4.97	0.240680
9	F2RL1	-8.28	0.323389
10	SOD1	-2.78	0.062629
12	HDAC2	-13.66	0.071392
14	SERPINA1	-7.96	0.089897
17	TGM2	-7.44	0.160842
20	HMOX1	-3.66	0.062155
22	RHOA	-12.50	0.145472
24	PPARG	-2.79	0.129727
26	CLDN1	-8.44	0.043282
27	STAT1	-6.51	0.332648
28	NFKBIA	-2.10	0.398001
30	TJP1	-11.70	0.350491
37	STAT3	-12.37	0.038667
38	APOA1	-10.94	0.102364
41	PPARA	-5.40	0.234438
42	NQO1	-2.71	0.755736
44	CCL20	-2.93	0.129089
47	RPLP0	-8.94	0.145134
